# SinusC-Net for automatic classification of surgical plans for maxillary sinus augmentation using a 3D distance-guided network

**DOI:** 10.1038/s41598-023-38273-9

**Published:** 2023-07-19

**Authors:** In-Kyung Hwang, Se-Ryong Kang, Su Yang, Jun-Min Kim, Jo-Eun Kim, Kyung-Hoe Huh, Sam-Sun Lee, Min-Suk Heo, Won-Jin Yi, Tae-Il Kim

**Affiliations:** 1 Department of Periodontology, School of Dentistry and Dental Research Institute, Seoul National University, Seoul, 03080 Republic of Korea; 2 Department of Biomedical Radiation Sciences, Graduate School of Convergence Science and Technology, Seoul National University, Seoul, 08826 Republic of Korea; 3 Department of Applied Bioengineering, Graduate School of Convergence Science and Technology, Seoul National University, Seoul, 08826 Republic of Korea; 4 Department of Electronics and Information Engineering, Hansung University, Seoul, 02876 Republic of Korea; 5 Department of Oral and Maxillofacial Radiology and Dental Research Institute, School of Dentistry, Seoul National University, Seoul, 03080 Republic of Korea

**Keywords:** Dental implants, Tomography

## Abstract

The objective of this study was to automatically classify surgical plans for maxillary sinus floor augmentation in implant placement at the maxillary posterior edentulous region using a 3D distance-guided network on CBCT images. We applied a modified ABC classification method consisting of five surgical approaches for the deep learning model. The proposed deep learning model (SinusC-Net) consisted of two stages of detection and classification according to the modified classification method. In detection, five landmarks on CBCT images were automatically detected using a volumetric regression network; in classification, the CBCT images were automatically classified as to the five surgical approaches using a 3D distance-guided network. The mean MRE for landmark detection was 0.87 mm, and SDR for 2 mm or lower, 95.47%. The mean accuracy, sensitivity, specificity, and AUC for classification by the SinusC-Net were 0.97, 0.92, 0.98, and 0.95, respectively. The deep learning model using 3D distance-guidance demonstrated accurate detection of 3D anatomical landmarks, and automatic and accurate classification of surgical approaches for sinus floor augmentation in implant placement at the maxillary posterior edentulous region.

## Introduction

When rehabilitating the posterior maxilla with implants, clinicians are frequently confronted with the problem of limited alveolar bone width and height. Sinus floor elevation via the lateral or transcrestal approach is the most predictable and commonly used method to resolve residual bone deficiency^1,2^. Several anatomic factors should be considered in selecting the surgical approach: residual bone height (RBH)^3^, sinus floor morphology^4,5^, presence of septa^6,7^, thickness of the lateral wall^8^, residual bone quality^9^, vascular anatomy^10^, and the number of teeth to be replaced. Among those factors, RBH is the most crucial factor in the decision-making process, and several classification systems or criteria were devised to select proper sinus elevation techniques based on RBH^3,11–13^.

Before placing implants at the posterior maxilla, clinicians find the ideal implant position and designate the landmarks in cone-beam computed tomography (CBCT) scans that are needed to measure RBH, horizontal bone width, and size of the vertical defect. Based on the preoperative evaluation, they determine the appropriate surgical approach. Fugazzotto proposed a protocol for selecting a surgical method that uses a mathematical formula for the RBH after reviewing treatment outcomes from the lateral or transcrestal approach with and without simultaneous implant placement^12^. Chiapasco et al. categorized maxillary alveolar bone defects into nine classes based on the width and height of the residual ridge and the relationship between the arches^3^. Unfortunately, those classifications are rarely used in treatment planning because they are excessively complex and detailed, and evaluation results are inconsistent among clinicians. The ABC sinus augmentation classification proposed by Hom–Lay Wang is a widely used, straightforward approach to treatment planning in the posterior maxilla^13^. The method broadly categorizes the edentulous posterior maxilla into three classes, A, B, and C, based on the location of the sinus floor relative to the alveolar bone crest. Then, it subdivides the classes based on amount of horizontal or vertical ridge resorption and suggests appropriate treatment options^13^.

Deep learning methods are widely used for detection^14–16^, classification^17–19^, segmentation^20–22^, and enhancement^23,24^ of medical and dental images. Deep learning has proved its worth in periodontology, including studies to identify periodontally compromised teeth^25^, detect alveolar bone loss^26,27^, and evaluate the severity of periodontitis^17,28^. In dental implant surgery, deep learning can assist clinicians by localizing critical anatomic structures such as the mandibular canals^29^ or measuring the ridge width and height from CBCT scans^30^. A recent study used deep learning to automatically detect missing teeth and to label the exact tooth number on CBCT images^31^. These achievements can save clinicians time and effort and increase the accuracy of implant surgery planning. In addition, deep learning can serve as validation for treatment outcomes by predicting implant stability or failure^32,33^.

To date, most deep learning research on treatment planning for implant placement has been limited to specific basic tasks, such as segmenting anatomic structures^29^ or identifying edentulous sites^31^. As far as we know, no previous studies have attempted to apply deep learning to surgical planning for maxillary sinus floor augmentation in implant surgery. In this study, we developed an automatic end-to-end method that used deep learning to replace the time- and labor-consuming process of determining the surgical approach for implant placement at the posterior maxilla.

We hypothesized that a deep learning model would be able to automatically determine the appropriate treatment plan of the maxillary posterior edentulous region in CBCT images according to the ABC sinus augmentation classification in implant placement^13^. Therefore, our objective in this study was to automatically classify surgical plans for maxillary sinus augmentation in implant placement at the maxillary posterior edentulous region using a 3D distance-guided network (SinusC-Net) that consisted of two stages: a volumetric regression network for anatomical landmark detection and a 3D distance-guided network for classifying the treatment plan. Our main contributions are as follows: (1) we applied convolutional long short-term memory (convLSTM)^34,35^, multi-scale inputs (MSI)^36^, and deep supervision (DS)^37,38^ in the volumetric regression network to accurately predict the locations of anatomical landmarks, and (2) we used the distance relationships among the predicted anatomical landmarks in 3D CBCT images as feature information that the 3D distance-guided network could use to determine the treatment plan.

## Materials and methods

### Data acquisition and preparation

This study was approved by the Institutional Review Board of Seoul National University Dental Hospital (No. ERI18001), and all data collection and experiments were conducted in compliance with relevant guidelines and regulations. The CBCT images were obtained from 133 patients (61 females and 72 males; mean age 62.65 ± 10.64 years) who visited Seoul National University Dental Hospital for implant treatment between November 2018 and March 2022. We included the patients’ CBCT images where the single tooth or two and three adjacent teeth were missing in the posterior maxilla, and those with the maxillary posterior edentulous area of which extraction sockets showed completed healing. We excluded the images with impacted third molars in the maxilla, anatomical abnormalities, or a history of surgery in the maxillary sinus, and those with invasive sinus pathology that required referral to an otolaryngologist, such as those with a likelihood of malignancy, opacifications exceeding $50{\%}$ of the sinus volume, air-fluid levels, or air bubbles^39^. Images of poor-quality due to severe artifacts from implants or metal restorations, and of a radiographic stent in place were also excluded.

We acquired patient data from three different CBCT systems. The CBCT images of 45, 45, and 43 patients were obtained using a Dinnova3 (HDXWILL, Seoul, Korea), CS 9600 (Carestream Health, Inc., Rochester, NY, USA), and CS 9300 (Carestream Health, Inc.), respectively. The CBCT images from the Dinnova3 were obtained under 100 kVp and 9 mA with a voxel size of 0.3 × 0.3 × 0.3 mm^3^, dimensions of 670 × 670 pixels, and 16-bit depth; those from the CS 9600 used conditions of 90 kVp and 8 mA with a voxel size of 0.15 × 0.15 × 0.15 mm^3^, dimensions of 1067 × 1067 pixels, and 16-bit depth; those from the CS 9300 used 80 kVp and 8 mA with a voxel size of 0.25 × 0.25 × 0.25 mm^3^, dimensions of 669 × 669 pixels, and 16-bit depth. We used cropped images of 256 × 256 × 250 pixels that were centered at the maxillary and mandibular regions. For deep learning, we prepared 72 volumes for the training dataset, 8 for the validation dataset, and 53 for the test dataset.

### Sinus augmentation classification and anatomical landmarks for classification

The ABC sinus augmentation classification method broadly divides the maxillary sinus into three classes (A, B, and C) based on residual bone height for deciding the surgical approach of maxillary sinus augmentation in implant placement at the posterior maxilla^13^. Class A represents abundant bone with a height greater than 10 mm below the sinus floor that allows proper implant placement. Class B indicates barely sufficient bone, with 6–9 mm of bone height beneath the sinus floor. Class C indicates compromised bone, with bone height of 5 mm or less below the sinus floor^13^. Cases that require additional grafting techniques along with sinus elevation are divided into sub-classifications: division h for horizontal defect, division v for vertical defect, and division c for combined defect^13^.

We modified the original ABC classification method into the simplified one consisting of five surgical approaches based on the study-specific criteria (Table 1) to effectively train the deep learning model, considering that all the sub-classifications required the guided bone regeneration (GBR). Three dentists with 2, 3, and 6 years of clinical experience evaluated and classified the 133 CBCT images according to our simplified method at the Department of Periodontology at Seoul National University Dental Hospital. The ground-truth class for each image was determined using a majority vote. After we determined the ground-truth classes, we calculated the Fleiss kappa value to evaluate inter-rater agreement and reliability^40^.

**Table 1:** The modified ABC classification for five surgical approaches to maxillary sinus augmentation.

Class	Diagnostic criteria	Recommended procedure(s)
A	RBH greater than 10 mm	Implant placement
B	RBH 6–9 mm	Osteotome
B’	RBH 6–9 mm with horizontal or vertical defect	Osteotome + GBR
C	RBH 5 mm or less	Lateral wall sinus elevation
C’	RBH 5 mm or less with horizontal or vertical defect	Lateral wall sinus elevation + GBR

*RBH* residual bone height, *GBR* guided bone regeneration.

**Figure 1:**
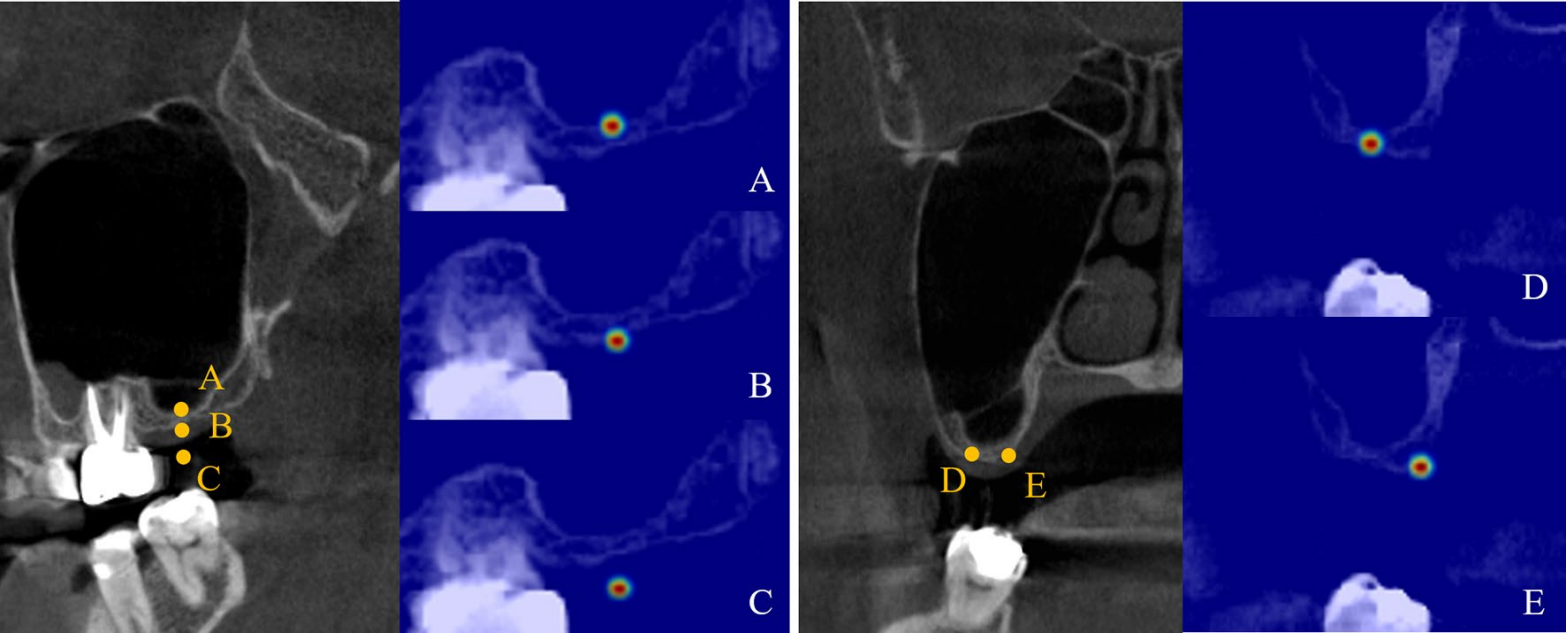
Five landmark locations and overlaid heatmaps in the sagittal (left) and coronal (right) planes in CBCT images. (A) maxillary sinus floor; (B) alveolar bone crest; (C) adjacent cementoenamel junction; (D) medial point of horizontal bone width; (E) lateral point of horizontal bone width.

The anatomical landmarks used in automatically classifying the maxillary sinus for the surgical approaches by deep learning consisted of the alveolar bone crest (AC), maxillary sinus floor (SF), medial point of the horizontal bone width (MH), lateral point of the horizontal bone width (LH), and adjacent cementoenamel junction (CEJ) (Fig. 1). One periodontist with 6 years of clinical experience annotated the landmarks on the CBCT volumes using a software (3D Slicer for Windows 10, Version 4.10.2; MIT, Massachusetts, USA)^41^.

### Overall framework for the deep learning model (SinusC-Net)

**Figure 2:**
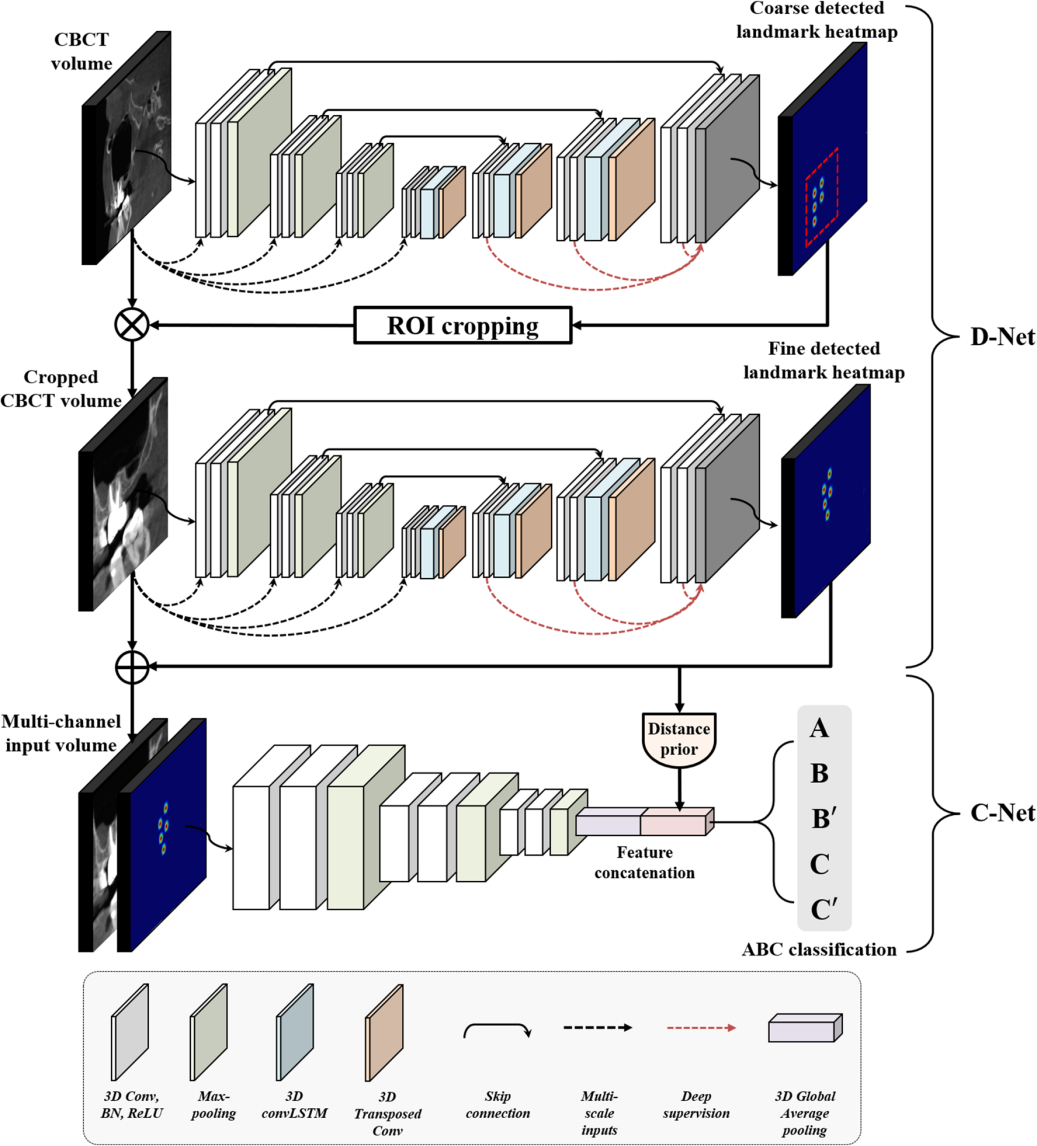
The overall framework of SinusC-Net, which consists of two stages: landmark detection using a cascaded volumetric regression network (D-Net) and classification according to the modified ABC classification on CBCT images using a distance-guided 3D network (C-Net).

The proposed framework for the deep learning model (SinusC-Net) consisted of two stages of detection and classification according to the modified ABC classification on CBCT images (Fig. 2). An overview of the SinusC-Net framework is presented in Fig. 2. In the first stage of anatomical landmark detection, the five landmarks were automatically detected in CBCT images using a cascaded volumetric regression network (D-Net) that relied on the 3D heatmap approach. The D-Net produced 3D heatmaps of the landmarks^42,43^, which were used as inputs alongside with the original CBCT images in the next stage. In the second stage of classification, a 3D distance-guided network (C-Net) uses the simplified ABC classes to automatically classify the CBCT images into the five surgical approaches.

To improve the landmark detection performance, we used 3D Gaussian heatmaps centered at the annotated landmark as labels (Fig. 1). Heatmap regression methods are widely used in recent advances in deep learning models for landmark localization tasks^42,43^. We used the Gaussian function to encode the distance matrix to obtain a normalized distance matrix as the 3D heatmap $H=\mathrm{exp}\left(-\frac{{D}^{2}}{2{\sigma }^{2}}\right)$, where $\sigma$ was a hyper-parameter for the width of the heatmap. In practice, since the 3D heatmap represented the probability of each landmark at each location, it could be considered a soft segmentation label, different from the labels used in general segmentation tasks. The soft segmentation allowed the network to robustly learn local geometric features from the neighboring points around each landmark^44^.

### Anatomical landmark detection network in SinusC-Net

We designed a cascaded network (D-Net) for accurate landmark detection using a coarse-to-fine learning strategy (Fig. 2). The main structure of the D-Net consists of 3D convolution blocks, 3D Max-pooling, 3D transposed convolution blocks, and skip connections. The 3D convolutional block includes 3 × 3 × 3 convolution, batch normalization, and rectified linear unit activation layers. Max-pooling and a 3D transposed convolution block were used for down-sampling and up-sampling, respectively, with a stride of two. Skip connections were used three times between an encoder and a decoder. The number of feature channels increased from 8 to 64 at each level of the layer. The sub-modules of D-Net consist of MSIs, convLSTM, and DS. To mitigate the loss of spatiotemporal information from the pooling layers, the MSI downsizes from the input volume using 2 × 2 × 2 average pooling, and it is concatenated at each level of the encoder^29^. The convLSTM is used to capture spatiotemporal information for learning the 3D anatomical structures of the input data^29^. The DS provides direct feedback to the hidden layers instead of only the final output layer by merging the feature maps from the different decoder layers^45^.

Using the coarse-to-fine learning strategy, the coarse D-Net was trained to detect coarse locations of the landmarks from CBCT volumes of 256 × 256 × 250 pixels. After training, the coarse D-Net produced coarse 3D heatmaps of the landmarks from the CBCT volumes. The volume for each landmark was then cropped into a volume of interest (VOI) patch of 128 × 128 × 128 pixels centered on the coarsely predicted location. For more accurate landmark detection, we trained the fine D-Net to regress the fine locations of the landmarks from the VOI patches. The coarse and fine D-Net were trained using cross-entropy (CE) loss with the DS approach. The CE was calculated as

$$CE\left(H,P\right)=-\frac{1}{n}\sum_{i}^{n} \left({H}_{i}\mathrm{log}\left({P}_{i}\right)+\left(1-{H}_{i}\right)\mathrm{log}\left(1-{P}_{i}\right)\right)$$

where $n$ is the number of pixels, $H$ is a 3D heatmap of the ground truth, and $P$ is a predicted 3D heatmap. To improve training stability and detection performance, the DS was used at each level of the decoder. In that way, the final loss function $(FL)$ based on the DS approach was determined as

$$FL\left(H,P\right)=C{E}_{1}\left(H,P\right)+C{E}_{2}\left(H,P\right)+C{E}_{3}\left(H,P\right)$$

where $C{E}_{l}$ is the loss calculated from each side output at the level of layer $l$ in the decoder. After training, the D-Net produced 3D heatmaps of the landmarks that were then used with the CBCT images as inputs for the C-Net.

### Sinus augmentation classification network of SinusC-Net

To determine the final classification, we designed the classification network (C-Net) using two-channel inputs, 3D convolution blocks, 3D Max-pooling, and distance priors (Fig. 2). The two-channel inputs of the original CBCT image and the corresponding 3D heatmap predicted by the D-Net were used to simultaneously learn both the anatomical structure and the geometric relationships between landmarks. The 3D convolutional block has 3 × 3 × 3 convolution, batch normalization, and rectified linear unit activation layers. 3D Max-pooling was used to down-sample the feature maps. To improve the distance awareness ability of the C-Net, we used three distance priors, which were the absolute distances between AC and SF, MH and LH, and AC and CEJ on the landmarks predicted by the D-Net. The three distance priors were concatenated with the flattened feature maps after 3D global average pooling. The C-Net was trained using the CE loss function defined above.

The networks were trained by the RMSprop optimizer for 300 epochs with an initial learning rate of 10^4^, which decreased by a factor of $0.5$ when the validation stopped decreasing for 25 epochs. We used a batch size of 8 and a single GPU with 24 Gb RAM. All networks were implemented in Python3 using a Keras framework with a Tensorflow backend. The data augmentation was performed with random rotation (in the range of − 25$^\circ$ to 25$^\circ$), re-scaling (in the range of – 10 to 20%), and modification of the intensity by randomly adjusting the brightness, contrast, saturation, and hue.

### Performance evaluation for detection and classification

We evaluated the predictive performance of the landmark localization by measuring the mean radial error (MRE) and the successful detection rate (SDR)^46^. The MRE was calculated as

$$\mathrm{MRE}={\sum }_{i}^{n}({R}_{i}/n)$$

where n is the number of data points, and R is the Euclidean distance between the center of ground truth and the predictive result. The SDR was calculated as the ratio at which the distance between the predicted and ground truth landmark was within a given distance (2.0 mm, 2.5 mm, 3.0 mm, and 3.5 mm).

To evaluate the classification performance, we calculated the accuracy ($ACC=\frac{TP+TN}{TP+TN+FP+FN}$), sensitivity ($Sens=\frac{TP}{TP+FN}$), specificity ($Spec=\frac{TN}{FP+TN}$), and area under the receiver operating characteristic (ROC) curve (*AUC*)^47^, where TP, TN, FP, and FN denote the true positive, true negative, false positive, and false negative, respectively.

Ablation studies were also conducted to evaluate impacts on the detection and classification performances by use of different modules (MSI, convLSTM, and DS) in detection network (D-Net) of SinusC-Net, and to evaluate impacts on the classification performance by use of distance priors in classification network (C-Net) of the SinusC-Net.

### Statistical analysis

To evaluate the inter-observer agreement in determining the ground truth of the ABC sinus augmentation classification from 133 CBCT scans, we applied Fleiss' kappa statistics using SPSS version 26 (SPSS Inc., IBM Corp.; Armonk, NY, USA). The statistical significance level was set to 0.05. The final classification results of the SinusC-Net were represented as a confusion matrix, from which we computed accuracy, sensitivity, and specificity. In addition, we calculated the AUC from the ROC curves, a graph that presents the true-positive rate in relation to false-positive rate by varying the discrimination threshold. We utilized Python (Python Software Foundation, Version 3.6.1; Wilmington, DE, USA) for the visualization and computation of the classification performance.

## Results

After we generated the ground truth classes according to the modified ABC classification, Fleiss kappa values were calculated to evaluate the consistency and reliability between observers (Table 2). The kappa value indicates the level of agreement: poor (0–0.2), fair (0.21–0.4), moderate (0.41–0.6), good (0.61–0.8), and almost perfect (0.81–1.0)^48^. The overall kappa value was 0.72 (P < 0.00), indicating that the three raters had good general consistency in determining the ground truth.

**Table 2:** The Fleiss kappa values for evaluating consistency and reliability between observers when determining the ground truth according to the modified ABC classification.

Class	Kappa	Standard error	Z-statistic	P value
A	0.91	0.05	18.18	0.00
B	0.59	0.05	11.69	0.00
B′	0.56	0.05	11.22	0.00
C	0.70	0.05	14.02	0.00
C′	0.77	0.05	15.71	0.00
Overall	0.72	0.03	28.24	0.00

**Figure 3:**
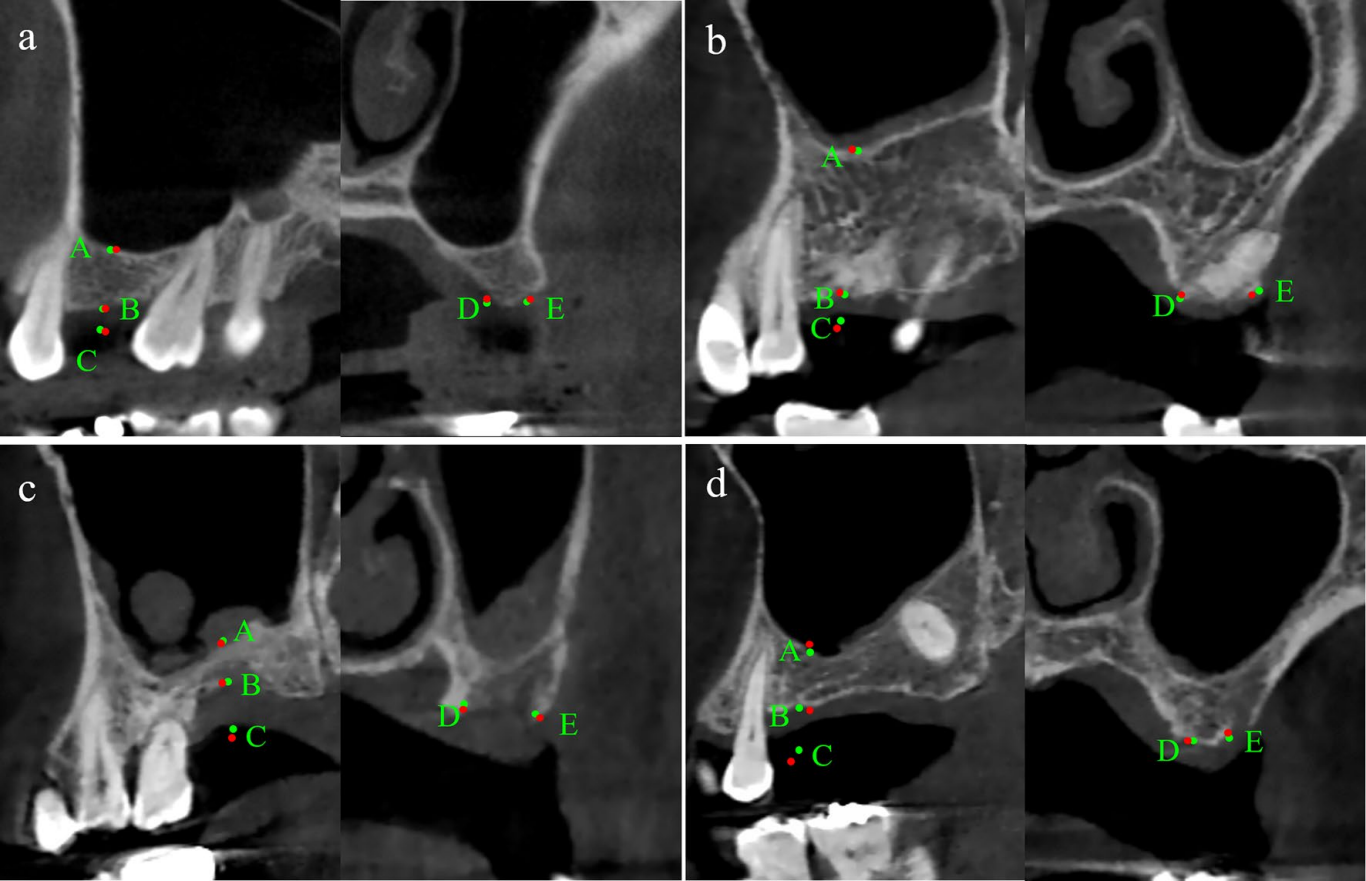
Landmark predictions in the sagittal (left) and coronal (right) planes (**a**–**d**). The predicted and ground truth landmarks are represented as red and green dots, respectively. (**a**) The best case with the least errors and (**d**) the worst case with the most errors. (A) Maxillary sinus floor; (B) alveolar bone crest; (C) adjacent cementoenamel junction; (D) medial point of horizontal bone width; (E) lateral point of horizontal bone width.

**Figure 4:**
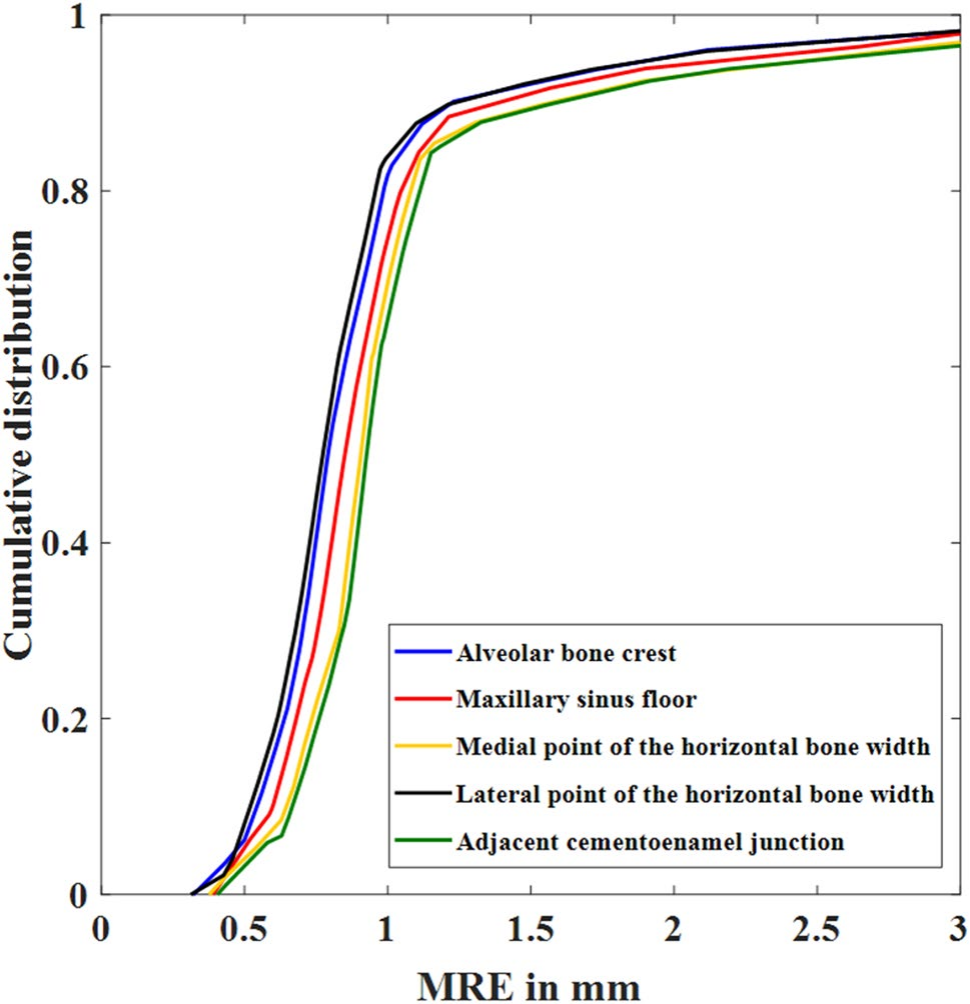
The cumulative distribution curves of MRE for five anatomical landmarks (alveolar bone crest, maxillary sinus floor, medial point of horizontal bone width, lateral point of horizontal bone width, and adjacent cementoenamel junction).

The anatomical landmark detection performance by the volumetric regression network (D-Net) of SinusC-Net was evaluated using the 53 CBCT volumes of the test dataset. The predicted and ground truth landmarks are represented with green and red dots, respectively, in Fig. 3. As can be seen in the figure, the D-Net of SinusC-Net performed well for landmark localization (Fig. 3). The SDR and MRE of the detection performance for the five anatomical landmarks in the test dataset are shown in Table 3. The mean MRE was 0.87 mm, and the SDR for 2 mm or lower was 95.47% for overall landmark detection. Among the anatomical landmarks, the lateral point of the horizontal bone width had the highest level of accuracy, with an MRE of 0.82 mm. On the other hand, the adjacent CEJ showed the lowest level of accuracy, with an MRE of 0.92 mm. The cumulative distribution curves of MRE show that the D-Net had high performance for all landmarks (Fig. 4). Therefore, the volumetric regression network of SinusC-Net achieved high performances for anatomical landmark detection, generally.

**Table 3:** The detection performance of five anatomical landmarks in terms of SDR and MRE by the detection network of SinusC-Net.

Anatomical landmark	SDR (%)			MRE (mm)
	< 2.0 mm	< 2.5 mm	< 3.0 mm	
Alveolar bone crest	96.23	98.11	98.11	0.85 $\pm$ 0.15
Maxillary sinus floor	96.23	96.23	98.11	0.88 $\pm$ 0.09
Medial point of the horizontal bone width	94.34	96.23	98.11	0.90 $\pm$ 0.13
Lateral point of the horizontal bone width	96.23	98.11	98.11	0.82 $\pm$ 0.07
Adjacent cementoenamel junction	94.34	96.23	96.23	0.92 $\pm$ 0.14
Mean (SD)	95.47 ± 0.93	96.98 ± 0.92	97.74 ± 0.75	0.87 $\pm$ 0.14

*SDR* successful detection rate, *MRE* mean radial error.

**Figure 5:**
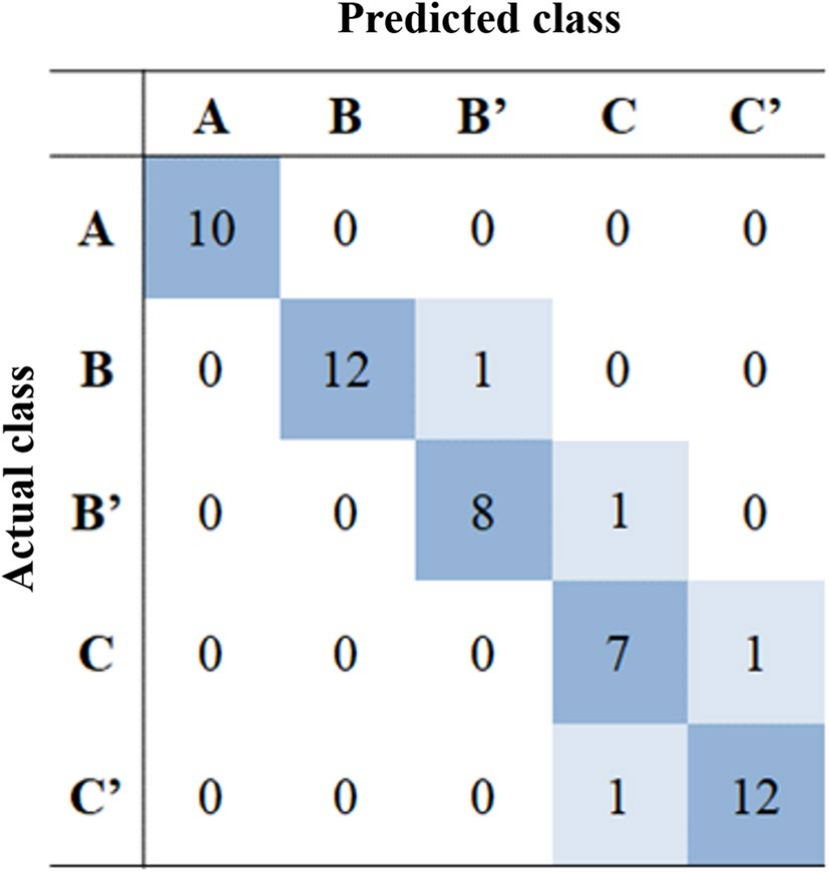
The confusion matrix for the classification results from SinusC-Net. The numbers represent the number of images classified correctly and incorrectly.

The classification performance by SinusC-Net was evaluated using the same 53 CBCT volumes of the test dataset. The confusion matrix presents the overall classification results for the five surgical approaches predicted by SinusC-Net (Fig. 5). The SinusC-Net achieved the performance of mean accuracy (ACC) of 0.97, sensitivity (Sens) of 0.92, specificity (Spec) of 0.98, and AUC of 0.95 (Table 4). Among the classes, Class A had the highest accuracy with an ACC of 1.00, Sens of 1.00, Spec of 1.00, and AUC of 1.00; and Class C had the lowest accuracy, with an ACC of 0.94, Sens of 0.92, Spec of 0.98, and AUC of 0.94. Thus, SinusC-Net showed the highest classification accuracy for Class A and the lowest for Class C, and its overall performance in classifying surgical approaches was high.

**Table 4:** The classification performance in terms of accuracy, sensitivity, specificity, and AUC by SinusC-Net.

Class	Accuracy	Sensitivity	Specificity	AUC
A	1.00	1.00	1.00	1.00
B	0.98	0.92	1.00	0.96
B’	0.96	0.89	0.98	0.93
C	0.94	0.88	0.96	0.92
C’	0.96	0.92	0.98	0.95
Mean (SD)	0.97 $\pm$ 0.02	0.92 $\pm$ 0.04	0.98 $\pm$ 0.01	0.95 $\pm$ 0.03

*AUC* area under the receiver operating characteristic curve.

**Figure 6:**
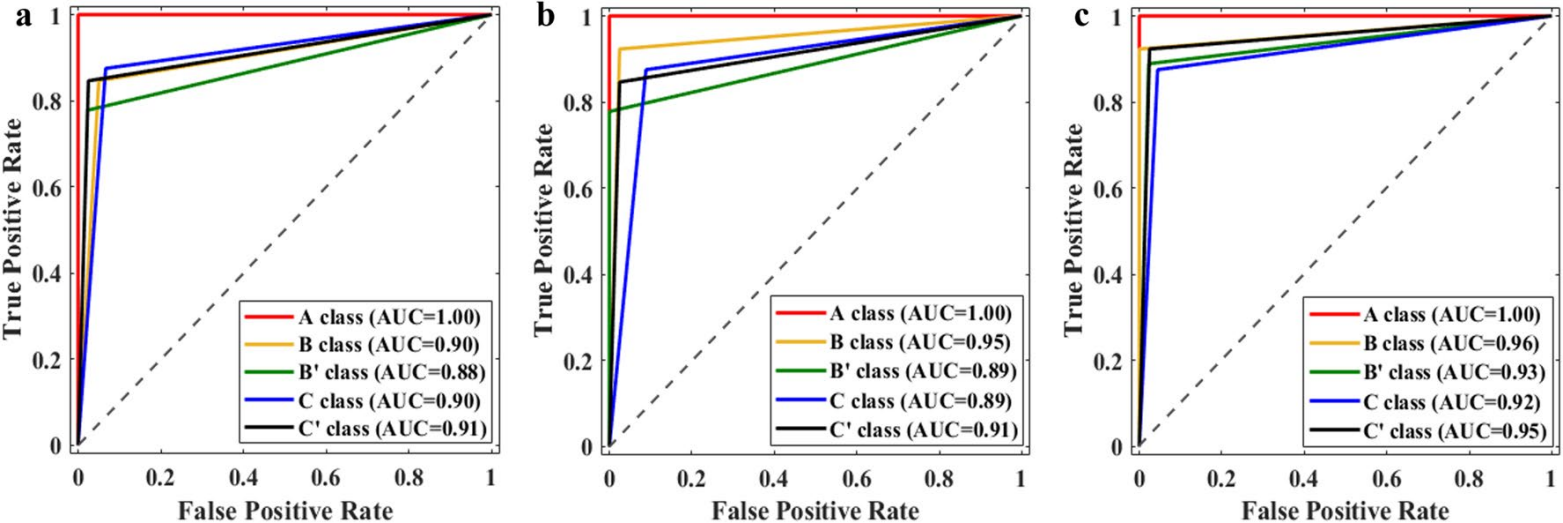
The ROC curves and AUC values for classification using D-Net with convLSTM (**a**), D-Net with convLSTM and MSI (**b**), and D-Net with convLSTM, MSI, and DS (SinusC-Net) (**c**). *convLSTM* convolutional long short-term memory, *MSI* multi-scale inputs, *DS* deep supervision.

The ablation study testing the use of different modules in the D-Net of SinusC-Net revealed that the network using both MSI and convLSTM was more accurate than that using only convLSTM, and the network using all three modules (convLSTM, MSI, and DS), which is the complete SinusC-Net, showed the highest performance in both detection and classification (Table 5). Furthermore, the ROC curve of the network using all three modules showed the highest AUC in classification (Fig. 6). Those results demonstrate the effectiveness of using convLSTM, MSI, and DS together in the detection and classification network of SinusC-Net. Additionally, the classification performance of the network using the distance priors was more accurate than that of the network not using them (Table 6), which demonstrates the effectiveness of applying distance priors for classification in SinusC-Net.

**Table 5:** Results of the ablation study using different modules in the detection network (D-Net) of SinusC-Net.

Module			Accuracy	Sensitivity	Specificity	SDR (< 2.0 mm)	MRE
convLSTM	MSI	DS					
✓			0.95 $\pm$ 0.02	0.87 $\pm$ 0.01	0.97 $\pm$ 0.02	93.12 $\pm$ 0.91	0.92 $\pm$ 0.11
✓	✓		0.95 $\pm$ 0.03	0.89 $\pm$ 0.02	0.97 $\pm$ 0.03	94.34 $\pm$ 0.89	0.90 $\pm$ 0.10
✓	✓	✓	0.97 $\pm$ 0.03	0.92 $\pm$ 0.02	0.98 $\pm$ 0.03	95.47 $\pm$ 0.93	0.87 $\pm$ 0.14

*convLSTM* convolutional long short-term memory, *MSI* multi-scale inputs, *DS* deep supervision, *SDR* successful detection rate, *MRE* mean radial error.

**Table 6:** Results of the ablation study on the use of distance priors in the classification network (C-Net) of SinusC-Net.

Model	Accuracy	Sensitivity	Specificity	AUC
Without distance priors	0.87 $\pm$ 0.02	0.87 $\pm$ 0.03	0.97 $\pm$ 0.04	0.88 $\pm$ 0.02
With distance priors	0.97 $\pm$ 0.03	0.92 $\pm$ 0.03	0.98 $\pm$ 0.04	0.95 $\pm$ 0.03

*AUC* area under the receiver operating characteristic curve.

## Discussion

In this study, we proposed a deep learning network with 3D distance-guidance (SinusC-Net) that can automatically classify surgical plans for sinus augmentation at the maxillary posterior edentulous region in CBCT images. SinusC-Net consists of a volumetric regression network for anatomical landmark detection (D-Net) and a 3D distance-guided network for classifying treatment plans (C-Net). The cascaded D-Net uses a coarse-to-fine learning strategy with MSI, convLSTM, and DS modules and demonstrated high accuracy in detecting the 3D anatomical landmarks used to determine treatment plans. For overall landmark detection by the D-Net, the MRE was 0.87 mm, and the SDR for 2 mm or lower was 95.47% of the time. The C-Net that used distance information between the landmarks as priors for classification demonstrated high classification accuracy that was consistent with the ground-truth results determined by clinicians. The mean accuracy, sensitivity, specificity, and AUC were 0.97, 0.92, 0.98, and 0.95, respectively, for the overall classification performance by SinusC-Net. We demonstrated the ability of the proposed deep learning network (SinusC-Net) to accurately and automatically classify surgical approaches for sinus floor augmentation in implant placement at the maxillary posterior edentulous region.

To date, many studies in dentistry have used deep learning models for automatic landmark detection to assist in orthodontic treatment or orthognathic surgery, mainly focusing on detecting landmarks on 2D lateral cephalometric images^49–51^. The MREs for landmark detection on 2D images by deep learning mostly ranged from 0.9 to 1.53 mm, and an SDR for 2 mm or lower from 77.01 to 82.43% of cases^49–51^. The accuracies of landmark detection on 3D images have been much lower than those on 2D images, with MREs of 3.63 to 5.785 mm^52,53^, because a large amount of volume data and high-performance hardware were required for the algorithms to learn 3D anatomical structures^52,53^. In the present study, to increase the landmark detection accuracy on 3D images, we used a cascaded volumetric regression network with a coarse-to-fine learning strategy. The first network produced coarse locations of the landmarks from whole CBCT volumes, and then smaller cropped VOI patches centered on the coarsely predicted landmarks were used for fine detection of the locations in the following network. Furthermore, our approach stands out from other methods because it incorporates convLSTM, MSI, and DS in constructing the detection network architecture. The use of those techniques in SinusC-Net enabled more accurate detection of 3D landmarks by learning the 3D anatomical structures of the input data through the convLSTM, and enhancing supervision of scaled features through MSI and DS.

The classification network used multi-channel inputs of the original CBCT image and the corresponding 3D heatmap predicted by the previous detection network to simultaneously learn both the anatomical structure and the geometric relationships between landmarks. Furthermore, to improve the distance awareness in the classification, three distance priors representing the absolute distances between the predicted landmarks were concatenated with the flattened feature maps after 3D feature encoding. Our results show the effectiveness of applying the distance priors to classification; SinusC-Net demonstrated high classification accuracy despite the small amount of data available. The distance-guided 3D network of the SinusC-Net had advantages in classification of surgical approaches according to the modified ABC classification, which were simultaneous learning of semantic relationships between anatomical structure and landmark distribution by using multi-channel inputs of the original CBCT image and the 3D heatmap of landmarks, and improving the distance awareness ability of the network using distance priors of the absolute distances between landmarks.

Although the deep learning model showed high classification accuracy for predicting surgical plans, the confusion matrix indicated that some of the predictions differed from the ground truth. The deep learning model showed perfect agreement between the predicted and actual classes in Class A cases, but disagreement occurred in other classes. However, that confusion was consistent with the clinicians' tendency in classifying the maxillary sinus when they were determining the ground truth. The individual kappa values indicated that the three clinicians showed high agreement for Class A and lower agreement for the other classes. In other words, the deep learning model shared the same difficulty with clinicians in classifying the maxillary sinus. Considering the deep learning model would also confuse with classes that would be ambiguous for clinicians, we modified the original ABC sinus augmentation classification by combining the sub-classifications when training the model. From a clinician’s point of view, it is often difficult to distinguish between horizontal, vertical, and combined bone deficiencies. Although we simplified the original classification, the recommended treatment approaches were the same because all the sub-classifications required GBR.

This study has some limitations. First, the deep learning model predicted classes consistent with the ground truth in cases in which the residual bone was clearly abundant, barely sufficient, or compromised, but it predicted incorrect classes in borderline cases. In cases of the residual bone on the borderline of the classes, clinicians choose an appropriate surgical approach by considering other factors, such as the anatomy of the maxillary sinus, patients' systemic conditions, and their own preference for less invasive techniques^54,55^. A more advanced deep learning model based on multi-modal inputs needs to be developed to incorporate data other than the residual bone volume in CBCT images so that it can provide more suitable treatment plans for borderline cases. Second, although we demonstrated that the deep learning model provided excellent outcomes in classifying surgical plans for maxillary sinus augmentation, the performance of our model needs to be validated for generalization and robustness. We used a relatively small sample of 133 CBCT images from three CBCT systems for our study due to our strict criteria for selecting images and the large size of data. Although we collected data from multiple CBCT machines to ensure model generalizability, we need to train and test the model using larger datasets from various organizations to improve its generalization.

Applying deep learning in treatment planning can help dental practitioners by enabling faster and more accurate treatment planning from precise measurements and automatic analyses of CBCT images. Deep learning techniques are particularly beneficial for treatment planning in digital dentistry because they can facilitate computer-aided diagnosis and design procedures with little to no manual intervention, producing significant advances in this field. Integrating deep learning research on surgical planning in the posterior maxilla with investigations on dental implant placement could automate the fabrication of surgical guides and enable optimal implant positioning in complicated cases.

## Conclusion

In this study, we proposed a deep learning network with 3D distance-guidance (SinusC-Net) to automatically classify surgical plans for maxillary sinus floor augmentation at the maxillary posterior edentulous region in CBCT images. SinusC-Net demonstrated accurate detection of 3D anatomical landmarks and automatic and accurate classification of surgical approaches for sinus floor augmentation in the maxillary posterior edentulous region during implant planning. These deep learning techniques can help dental practitioners by enabling faster and more accurate treatment planning with precise measurements and automatic analysis of CBCT images in digital dentistry.

## Data Availability

The datasets generated and/or analyzed during the current study are not publicly available due to restriction by the Institutional Review Board of Seoul National University Dental Hospital to protect patient privacy, but they are available from the corresponding author upon reasonable request. Please contact the corresponding author for any commercial implementation of our research.
